# Long‐term adherence to lifestyle changes and association with cognitive change: 11‐year results from the FINGER randomized, controlled trial

**DOI:** 10.1002/alz70860_106542

**Published:** 2025-12-23

**Authors:** Tiia Ngandu, Alina Solomon, Jenni Lehtisalo, Riitta Antikainen, Tuomo Hänninen, Tiina Laatikainen, Esko Levälahti, Jaana Lindstrom, Francesca Mangialasche, Teemu Paajanen, Markku Peltonen, Hilkka Soininen, Timo Strandberg, Jaakko Tuomilehto, Miia Kivipelto

**Affiliations:** ^1^ Finnish Institute for Health and Welfare, Helsinki, Finland; ^2^ University of Eastern Finland, Kuopio, Finland; ^3^ Imperial College London, London, United Kingdom; ^4^ Karolinska Institutet, Stockholm, Sweden; ^5^ University of Oulu, Oulu, Finland; ^6^ Oulu University Hospital, Oulu, Finland; ^7^ Kuopio University Hospital, Kuopio, Finland; ^8^ Karolinska University Hospital, Stockholm, Sweden; ^9^ Finnish Institute of Occupational Health, Helsinki, Finland; ^10^ University of Helsinki, Helsinki, Finland; ^11^ South Ostrobothnia Central Hospital, Seinäjoki, Finland; ^12^ FINGERS Brain Health Institute, Stockholm, Sweden

## Abstract

**Background:**

The Finnish Geriatric Intervention Study to Prevent Cognitive Impairment and Disability (FINGER) previously showed that a 2‐year multidomain lifestyle intervention has a beneficial effect on cognitive function among at‐risk older adults. Furthermore, both participation in the intervention activities and the lifestyle changes achieved were associated with more improvement in cognition. We investigated the long‐term effects of the intervention on lifestyle and cognition over 11 years.

**Method:**

FINGER included 1259 individuals, aged 60‐77 years with an increased risk of dementia. The participants were randomized to receive either regular health advice (control) or an intervention comprising of exercise training, dietary counselling, cognitive training, and management of vascular risk factors (intervention) lasting for 2 years. Participants were invited to follow‐up visits approximately 5, 7 and 11 years after the baseline visit. Lifestyles were measured with an index comprising self‐reported questions on diet, physical activity, cognitive and social activities, smoking and alcohol use. The participants were categorized into 4 groups based on their engagement in intervention activities: the control group; the intervention group with low participation; the intervention group with intermediate participation; and the intervention group with high participation. Neuropsychological test battery composite score was applied to measure cognition.

**Result:**

The beneficial effect of intervention on lifestyles was sustained until 7 years after baseline (*p* = 0.005 for intervention vs. control at 7 years). The lifestyles were maintained better among older participants and those with high participation during the intervention period, compared with the control group throughout the entire 11 y period. Early drop‐out from the study was linked to worse lifestyles. Overall, cognitive performance level increased until 2 years and declined thereafter. Individuals with high intervention engagement had better cognitive trajectories compared with the control group until 7 years (*p* = 0.003) and compared with those with low engagement until 11 years (*p* = 0.002).

**Conclusion:**

This study provides the first evidence that multidomain, lifestyle‐based interventions lasting for 2 years can have beneficial effects on lifestyles and cognition even several years after the intervention. Especially participants who adhered well to the intervention had sustained benefits. These findings further support the implementation of such preventive activities.

